# 

**Published:** 1905-10

**Authors:** 


The Great American Blight.

Vol. XXI. OCTOBER, 1905. No. 4.

### TEXAS Medical Journal.

TWENTY-FIRST YEAR.

Subscription, $1.00 a Year, in Advance.
Single Copies, 10 Cents.

PUBLISHED AT AUSTIN, TEXAS, BY

F. E. DANIEL, M. D.,

Proprietor.

PRINTED BY THE VON BOECKMANN-JONES CO.

Entered at the Postoffice at Austin, Texas, as Second-class Mail Matter.

